# C–N Coupling of 3‐Aminothiophene with Substituted Benzenediazonium Cations: Carbon Nucleophilicity, Hyper‐Ortho Effects, and Predictive Modeling of Structural Analogs

**DOI:** 10.1002/cphc.202500553

**Published:** 2025-11-03

**Authors:** R. El Abed, T. Slama, F. Mahdhaoui, T. Boubaker

**Affiliations:** ^1^ Laboratoire de Chimie Hétérocyclique, Produits Naturels et Réactivité (LR11SE39), Faculté des Sciences de Monastir Université de Monastir Avenue de l’Environnement 5019 Monastir Tunisie

**Keywords:** 3‐aminothiophene, kinetics, Mayr–Patz reactivity scale, nucleophilicity, structure–reactivity relationships, substituent electronic effects

## Abstract

This review has kinetically investigated the electrophilic attack of 3‐aminothiophene **1** by a series of *para*‐substituted benzenediazonium cations **7a–7h** in 50% H_2_O‐50% Me_2_SO at 20 °C using stopped‐flow spectrophotometry. No kinetic isotope effect is observed with the 2‐deuterio‐3‐aminothiophene, confirming that the rate‐determining step is a carbon‐based electrophilic aromatic substitution (S_E_Ar) at the C–2 position. The Hammett plot with *σ*_p_ values shows nonlinearity due to electron‐donating substituents. However, a linear relationship is obtained using the Yukawa–Tsuno equation, highlighting the resonance contribution via the r(*σ*_p_^+^ − *σ*_p_) term. An excellent linear correlation (*R* ^2^ ≈ 0.9968) is observed between log *k*_1_ and the experimental electrophilicity parameter ***E*** of the diazonium cations, as defined in the Mayr–Patz equation, allowing the determination of the carbon nucleophilicity parameters of 3‐aminothiophene: *N* = 9.37 and s_N_ = 1.18. Importantly, a strong linear relationship is established between *N* and the Hammett *σ* ^+^ constants for 3‐substituted 3‐aminothiophenes (*R* ^2^ = 0.9763), described by the equation: *N* = 6.72 – 2.01 *σ* ^+^. This correlation not only demonstrates the pronounced enaminic behavior of 3‐aminothiophenes but also enables the prediction of *N* values for unmeasured analogs, confirming that substituent–*π*‐system interactions govern nucleophilic reactivity via a hyper‐ortho electronic effect.

## Graphical abstract

The nucleophilicity parameters of 3‐aminothiophene (*N* = 9.37; s_N_ = 1.18) are determined using the Mayr–Patz equation. A linear correlation with Hammett *σ* ^+^ constants (*N* = 6.72 – 2.01 *σ* ^+^) is established, enabling prediction of nucleophilic reactivity for 3‐substituted derivatives in 50% DMSO–50% H_2_O at 20 °C.

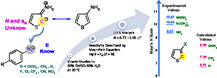

## 1 Introduction

Although 3‐aminothiophene **1** and aniline display comparable amino basicities (pK_a_ = 3.38^[^ ^1^ ^]^ and 4.20^[^ ^2^ ^]^ in water, respectively), their nucleophilic reactivities toward electrophiles differ markedly. The reference reaction with 4,6‐dinitrobenzofuroxane (DNBF) **2**, a well‐known super‐electrophile used to probe weak nucleophiles,^[^ ^3^, ^4^, ^5^, ^6^, ^7^, ^8^ ^]^ highlights this difference. In the case of aniline, Buncel and coworkers^[^ ^2^ ^]^ showed that the first attack takes place at the nitrogen atom, giving the *σ*‐adduct **3** with a C—N bond, in line with the usual reactivity of amines. Since **3** is less stable than its carbon analog, it slowly dissociates and finally produces the more stable carbon‐bonded complex **4** with a C—C bond as the only final product. In contrast, Terrier and coworkers^[^ ^1^ ^]^ found that the reactions of 3‐aminothiophene **1** with **2** lead exclusively to C‐bonded *σ*‐adducts **5**, while the expected nitrogen *σ*‐complex **6** was never observed under any experimental conditions, either in water or in H_2_O–Me_2_SO mixtures.


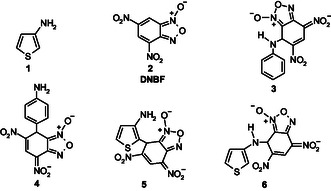


The nonreactivity of 3‐aminothiophene **1** towards nitrogen with various electrophilic motifs has also been observed by synthetic organic chemists.^[^ ^9^ ^]^ This property is indeed very important because the predominance of C‐nucleophilicity in 3‐aminothiophene **1** allows for the easy synthesis of numerous thiophene derivatives with interesting pharmacological properties.^[^ ^10^, ^11^ ^]^ Fundamentally, this behavior appears to arise from the pronounced enaminic character of 3‐aminothiophene **1** and its structural analogs.

**Scheme 1:**
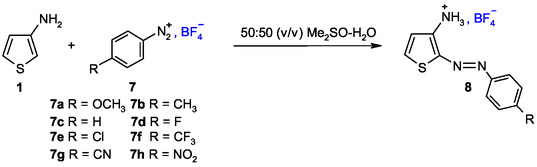
Carbon‐nitrogen coupling reactions of 3‐aminothiophene **1** with *para*‐R‐substituted benzenediazonium cations **7** in a 50% H_2_O‐50% Me_2_SO mixture. Products **8** are represented in the (E)‐configuration, which has recently been reported as the thermodynamically most stable form.^[^ ^12^ ^]^

Based on these results, it became clear that additional information on the behavior of aminothiophene **1** was needed. Specifically, its reactivity with other electrophiles had to be investigated to allow for kinetic quantification. As a result, we conducted a detailed study of the C–N coupling reaction between aminothiophene **1** and various *para*‐R‐benzenediazonium cations **7a–7h** in a 50% H_2_O‐50% Me_2_SO mixture at 20 °C (**Scheme** **1**). This study provided the kinetic data required to quantify its carbon nucleophilicity and to assess the influence of substituent effects on reaction rates through Hammett‐type analysis. By combining our data with previously reported results by Terrier and coworkers on other 3‐substituted aminothiophenes, we established a strong linear correlation between the nucleophilicity parameter *N* and the Hammett *σ* ^+^ constants of C‐3 substituents. As we will see, the relationship established between the nucleophilicity parameter *N* and the *σ* ^+^ constants of C‐3 substituents revealed a hyperortho electronic effect arising from substituent–*π*‐system interactions and further enabled the prediction of *N* values for structurally related analogs within the thiophene series.

## 2 Results and Discussion

### 2.1 Kinetic Investigations: Reactions of 3‐Aminothiophene 1 with Cations 2

**Figure 1:**
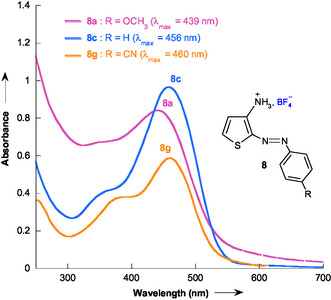
UV–vis absorption spectra at the end of the reaction for the 3‐aminothiophene–benzenediazonium systems **7a**, **7c**, and **7g** at pH = 1 in 50% H_2_O‐50% Me_2_SO v/v at 20 °C.

The diazo C‐adducts **8a–8 h**, generated by the in situ coupling of benzenediazonium cations **7a–7h** with 3‐aminothiophene **1**, display UV–vis absorption maxima in the 439–498 nm range. As an illustrative case, **Figure** **1** shows the absorption spectrum recorded at the end of the reaction for the 3‐aminothiophene–benzenediazonium systems **7a**, **7c**, and **7g** at pH = 1. Notably, Terrier and coworkers^[^ ^1^ ^]^ reported similar absorption maxima (*λ*_max_ ≈ 480 nm) for the *σ*‐adducts **5** formed between 4,6‐dinitrobenzofuroxan **2** and 3‐aminothiophene **1** in 50:50 v/v Me_2_SO‐H_2_O, which fall within the same spectral region (≈439–498 nm). Despite differences in their *π*‐systems, the C‐adducts and *σ*‐adducts exhibit visible absorption in a similar range, allowing convenient monitoring of the reaction by UV–vis spectroscopy.

In most cases, our experiments were conducted using a stopped‐flow spectrophotometer. To simplify the kinetic equations, all measurements were performed with concentrations of 3‐aminothiophene **1** (ranging from 10^−3^ to 2.80 × 10^−2^ mol L^−1^) significantly higher than those of the cations **7a–7h** (≈5 × 10^−5^ mol L^−1^) in the Me_2_SO‐H_2_O 50%‐50% v/v medium. This allowed us to operate under pseudo‐first‐order conditions, favorable for the analysis of the kinetic data collected. The reactions were studied at 20 °C at pH = 2 and pH = 1, using hydrochloric acid solutions. The ionic strength of the medium was kept constant at 0.1 mol L^−1^ by adding potassium chloride.

**Scheme 2:**
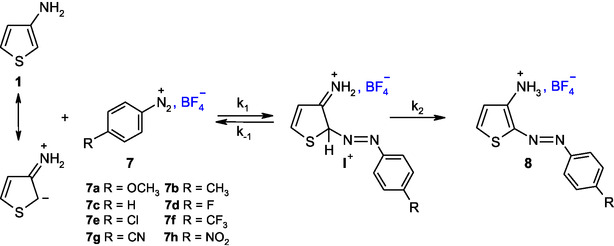
Carbon‐nitrogen coupling reactions of 3‐aminothiophene **1** with *para*‐R‐substituted benzenediazonium cations **2** in 50% H_2_O‐50% Me_2_SO v/v.

In all experiments conducted, spectroscopic monitoring revealed a single relaxation process. By analogy with the coupling mechanism proposed in the literature for the reactions of various carbon nucleophiles with superelectrophiles,^[^ ^12^, ^13^, ^14^, ^15^, ^16^ ^]^ it is reasonable to assume that the diazo adducts **8a–8h** formed with 3‐aminothiophene **1** result from an electrophilic aromatic substitution (S_E_Ar) occurring at the C‐2 position of the nucleophile, via the formation of a Wheland‐type *σ*‐complex intermediate. As depicted in **Scheme** **2**, the diazo adducts **8a–8h** arise from a two‐step S_E_Ar pathway involving an initial addition step followed by elimination. Kinetically, the bimolecular rate constant *k*_1_ corresponds to the addition of benzenediazonium salts **7a–7h** to aminothiophene **1**, while the unimolecular rate constants *k*_−1_ and *k*_2_ describe the decomposition of the *σ*‐complex intermediate **I** ^**+**^, either by reversion to the starting materials or by re‐aromatization of the thiophene ring to yield the final products.

In this context, the observed pseudo‐first‐order rate constant (*k*_obsd_) for the formation of diazo adducts **8a–8h** at a given pH can be determined by applying the quasi‐steady‐state assumption to the intermediate complex **I** ^**+**^, a thermodynamically unstable species. This approach leads to the following relation (1).^[^ ^1^ ^]^

(1)

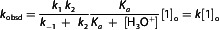




**Figure 2:**
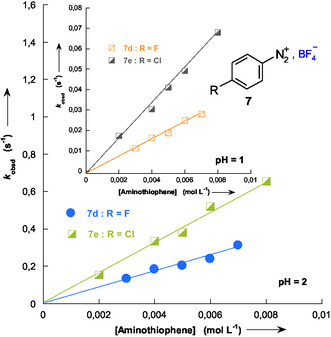
Influence of the total concentration of 3‐aminothiophene **1** and pH on the pseudo‐first‐order rate constants (*k*_obsd_) for its addition to benzenediazonium salts **7d** and **7e** in 50% H_2_O–50% Me_2_SO v/v at 20 °C. Reactions were monitored at pH 1 and 2.

In agreement with this expression, excellent linear correlations were observed at each pH studied when the pseudo‐first‐order rate constants (*k*_obsd_) were plotted as a function of the total concentration of aminothiophene **1**, as illustrated in **Figure** **2**. Additional kinetic data obtained under various conditions are provided in Table S1–S11 and Figure S1–S9, Supporting Information.

**Table** **1** summarizes the values of the second‐order rate constants *k*, along with those obtained from a series of experiments involving 2‐deuteriated‐3‐aminothiophene. Notably, the data presented in Table 1 show that isotopic substitution at C‐2 does not significantly affect the rate of formation of adducts **8a–8h**. Indeed, the measured *k* ^H^/*k* ^D^ ratios are found in the range 0.96–1.14, indicating that the nucleophilic addition is the rate‐determining step, i.e., *k*_2_ >> *k*_−1_ in Scheme 2. Accordingly, the observed pseudo‐first‐order rate constant (*k*_obsd_) reduces to the simplified expression given in Equation (2), from which the values of *k*_1_ for the addition of **1** to the benzenediazonium cations **7a–7h** were determined at each pH (see Table 1).
(2)

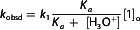




**Table 1:** Effect of pH and substituent nature on the bimolecular rate constants (*k*) and relative rate constants (*k*_1_) for the coupling of 3‐aminothiophene **1** with benzenediazonium cations **7a–7h** in 50% H_2_O–50% Me_2_SO v/v at 20 °C and I = 0.1 mol L^−1^.

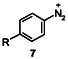		*k* a) M^−1^ s^−1^	*k*_1_ b) M^−1^ s^−1^	*k* a) M^−1^ s^−1^	*k*_1_ b) M^−1^ s^−1^	*σ*_p_ d)	*σ*_p_^+^ d)	*E* e)
		pH = 2		pH = 1				
**7a**	R = OCH_3_	5.30 × 10^−1^ (4.90 × 10^−1^)c)	1.33 × 10^1^ (1.23 × 10^1^)c)	6.00 × 10^−2^ (5.30 × 10^−2^)c)	1.45 × 10^1^ (1.30 × 10^1^)c)	−0.27	−0.78	−8.4
**7b**	R = CH_3_	2.78	6.95 × 10^1^	2.40 × 10^−1^	5.88 × 10^1^	−0.17	−0.31	−7.7
**7c**	R = H	1.71 × 10^1^ (1.79 × 10^1^)c)	4.27 × 10^2^ (4.45 × 10^2^)c)	1.93 (1.67)^c)^	4.65 × 10^2^ (4.12 × 10^2^)c)	0	0	−7.2
**7d**	R = F	4.12 × 10^1^	1.03 × 10^3^	4.19	1.01 × 10^3^	0.06	−0.07	–
**7e**	R = Cl	8.46 × 10^1^	2.11 × 10^3^	8.54	2.06 × 10^3^	0.23	0.11	−6.7
**7f**	R = CF_3_	1.03 × 10^3^	2.57 × 10^4^	1.07 × 10^2^	2.58 × 10^4^	0.54	0.61	−5.7
**7g**	R = CN	1.38 × 10^3^ (1.21 × 10^3^)c)	3.44 × 10^4^ (3.02 × 10^4^)c)	1.29 × 10^2^ (1.31 × 10^2^)c)	3.10 × 10^4^ (3.21 × 10^4^)c)	0.66	0.66	−5.5
**7h**	R = NO_2_	3.83 × 10^3^	9.55 × 10^4^	3.45 × 10^2^	8.32 × 10^4^	0.78	0.79	−5.1

a) Values of *k* were determined in this work from the slope of plots of *k*_obsd_ *vs*. [1]_o_ in 50% H_2_O–50% Me_2_SO v/v (for details, see Table S1–S7, Supporting Information).

b) Rate constants *k*_1_ were calculated in this work by using *k*, [H_3_O^+^], and the K_a_ = 10^−3.38^ for aminothiophene **1** taken from ref. [1].

c) Value for the corresponding 2‐deuterio‐3‐aminothiophene.

d) *σ*_p_ and *σ*_p_^+^ values were taken from ref. [33].

e) Electrophilicity parameters *E* taken from ref. [24].

### 2.2 Electronic Effects: Insights from Hammett and Yukawa–Tsuno Correlations

Analysis of the data presented in Table 1 clearly reveals a strong influence of the *para*‐substituent R on the reactivity of benzenediazonium salts toward 3‐aminothiophene **1**. The bimolecular rate constants (*k*_1_) decrease markedly as the electron‐withdrawing ability of R diminishes. For example, at pH = 1, *k*_1_ decreases from 8.32 × 10^4^ M^−1^ s^−1^ for R = NO_2_ to 1.45 × 10^1^ M^−1^ s^−1^ for R = OCH_3_. This trend highlights the crucial role of electronic effects in modulating the electrophilic character of the diazonium salts.

**Figure 3:**
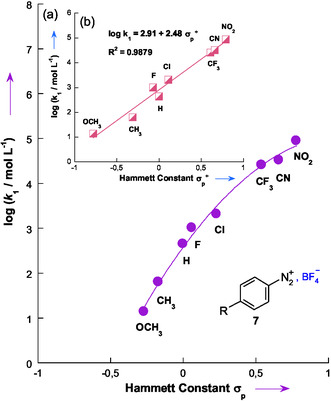
Hammett plots of log k_1_ for the coupling reactions of 3‐aminothiophene **1** with benzenediazonium salts **7a–7h** in 50% H_2_O–Me_2_SO v/v at 20 °C using a) *σ*_p_ and b) *σ*_p_^+^ Hammett constants.

To quantify this influence, we first examined the correlation between the logarithm of the rate constants (log *k*_1_) and the Hammett substituent constants. As depicted in **Figure** **3**, the plot of log *k*_1_ versus *σ*_p_ shows substantial deviation from linearity, particularly for electron‐donating substituents such as **CH_3_** and **OCH_3_**, which exhibit significantly lower reactivities than predicted by their *σ*_p_ values. This observation indicates that classical inductive effects alone are insufficient to account for the observed substituent‐dependent reactivity. In contrast, a significantly improved linear correlation is observed when using the *σ*_p_^+^ constants (Figure 3), which incorporate both inductive and resonance contributions. The best‐fit regression line is described by Equation (3).

(3) $$\text{log }k_{1}=2.91+2.48\;\sigma_{p}^{+}\;(R^{2}=0.9879)$$

The high Hammett reaction constant (*ρ* = 2.48) reflects a strong sensitivity of the reaction rate to the electronic nature of *para*‐substituents on the electrophilic partner. This substantial *ρ* value clearly indicates significant positive charge development on the nucleophilic moiety already in the transition state. Interestingly, this value is comparable to that reported for the C–N coupling of 3‐methoxythiophene with benzenediazonium salts (*ρ* = 2.11),^[^ ^15^ ^]^ a well‐documented reaction that is highly responsive to substituent effects. These findings support a mechanism in which the rate‐determining step involves nucleophilic attack on an electron‐deficient diazonium center, with the process being strongly accelerated by electron‐withdrawing groups through inductive stabilization of the transition state.

**Figure 4:**
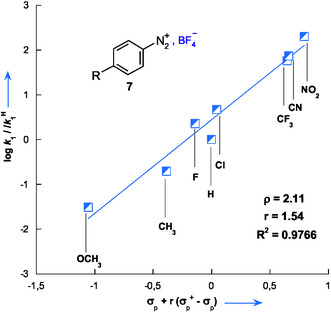
Yukawa–Tsuno correlation for reactions of 3‐aminothiophene **1** with benzenediazonium salts **7a–7h** in 50% H_2_O–50% Me_2_SO v/v at 20 °C. The *σ*_p_ and *σ*_p_^+^ values were taken from ref. [33].

To further assess the role of resonance effects in modulating the reactivity of benzenediazonium salts, the experimental data were analyzed using the Yukawa–Tsuno formalism.^[^ ^17^, ^18^ ^]^ While the classical Hammett plot^[^ ^19^ ^]^ shows notable deviations from linearity, the corresponding Yukawa–Tsuno correlation provided a satisfactory linear fit (**Figure** **4**), yielding a reaction constant *ρ* = 2.11 and a resonance parameter *r* = 1.54.

These results indicate a significant contribution of resonance effects, particularly for electron‐donating substituents such as 4‐methoxy and 4‐methyl, which show negative deviations from the Hammett line. This behavior can be attributed to ground‐state stabilization via mesomeric interactions of the methoxy group and hyperconjugative effects of the methyl group with the diazonium moiety.^[^ ^20^, ^21^, ^22^ ^]^ This interpretation is supported by the satisfactory Yukawa–Tsuno linear fit, which explicitly includes a resonance parameter (*r*) to quantify the contribution of substituent conjugation to the observed reactivity trend.

### 2.3 Quantifying the C‐Nucleophilicity of 3‐Aminothiophene

To investigate the influence of electrophilic strength on the reactivity of cations **7a**–**7h**, we apply the Mayr–Patz Equation (4)^[^ ^23^ ^]^ to quantify the nucleophilicity at C‐2 position of 3‐aminothiophene **1**, based on its second‐order rate constants with a series of reference diazonium salts of known electrophilicity^[^ ^24^ ^]^ (see Table 1).

(4) $$\text{log }k=s_{N}\left(E+N\right)$$

**Figure 5:**
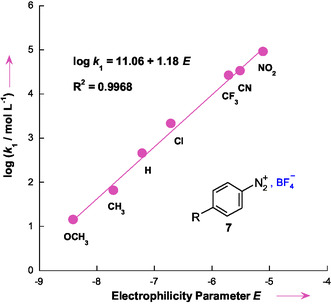
Plot of the rate constants (log *k*_1_) for the reactions of 3‐amonothiophene **1** with reference electrophiles **7a–7h** in 50% H_2_O–50% Me_2_SO v/v at 20 °C versus their electrophilicity parameters *E.*

In this equation, *N* and s_N_ are nucleophile‐specific parameters, and *E* is an electrophile parameter. As shown in **Figure** **5**, an excellent linear correlation was obtained by plotting log *k*_1_ against the electrophilicity values *E* of benzenediazonium salts **7a–7h**, in accordance with Equation  (5).

(5) $$\text{log }k_{1}=11.06+1.18\;E\;\;\;(R^{2}=0.9968)$$

From the slope and intercept, the nucleophilicity parameters of 3‐aminothiophene were determined to be *N* **= **9.37 and s_N_ = 1.18. These values indicate that 3‐aminothiophene behaves as a relatively strong carbon nucleophile, exhibiting moderate sensitivity to changes in the electrophilic nature of the reaction partner.

The experimentally determined s_N_ = 1.18 for 3‐aminothiophene reflects a moderate to high sensitivity to the electrophilic partner, indicating a notable degree of polarizability at the nucleophilic site. This value places 3‐aminothiophene in a similar range as conjugated carbon nucleophiles such as 3‐methoxythiophene (s_N_ = 1.19, acetonitrile),^[^ ^25^ ^]^ 5‐methoxyindole (s_N_ = 1.12, dichloromethane),^[^ ^13^ ^]^ and 1,3‐dimethoxybenzene (s_N_ = 1.09, dichloromethane).^[^ ^26^ ^]^ The observed similarity indicates that the conjugation between the NH_2_ group and the thiophene ring efficiently stabilizes the charge development in the transition state, which in turn increases the site's electronic reactivity.

Compared with other π‐rich heterocycles such as pyrrole (s_N_ = 1.00),^[^ ^27^ ^]^ and *N*‐methylpyrrole^[^ ^26^ ^]^ (s_N_ = 1.03), 3‐aminothiophene displays slightly greater sensitivity, which likely arises from more efficient delocalization of the amino lone pair into the thiophene ring. In contrast, cyclic enamines such as 1‐(*N*‐piperidino)cyclohexene (s_N_ = 0.83),^[^ ^28^ ^]^ and 1‐(*N* **‐**morpholino) cyclohexene (s_N_ = 0.81)^[^ ^28^ ^]^ show significantly lower sensitivities.

### 2.4 Validation and Comparative Nucleophilicity Analysis

The nucleophilicity parameter (*N*) of 3‐aminothiophene **1** was validated by reference to the well‐established nucleophilic aromatic substitution of 4,6‐dinitrobenzofuroxan (DNBF, **2**), previously investigated in detail by Terrier and coworkers.^[^ ^1^ ^]^ The corresponding second‐order rate constant under comparable conditions was analyzed using Equation (4), with the electrophilicity parameter of DNBF (*E* = −5.06).^[^ ^29^ ^]^ The value obtained is summarized in **Table** **2**. Importantly, the *N* value estimated for 3‐aminothiophene **1** (*N* = 9.59) closely matches the experimentally determined value (*N* = 9.37), thus providing strong support for the reliability of the nucleophilicity parameter derived in this work.

**Table 2:** Comparative study of nucleophilicity parameters *N* in 50% H_2_O–50% Me_2_SO v/v for selected C‐nucleophiles including pyrroles **11** and **12**, indoles **13** and **14**, and 3‐aminothiophenes **1**, **9**, and **10**. The reference electrophile is 4,6‐dinitrobenzofuroxan **2** with *E* = –5.06.^[^ ^29^ ^]^

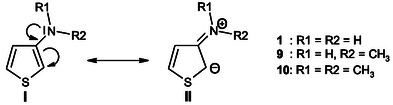		
Nucleophile	k (*M* ^−1^ s^−1^)a)	Nucleophilicity parameter Nb)
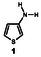 3‐Aminothiophene	3.37 × 10^4^	9.59 (9.37)c)
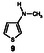 3‐(Methylamino)thiophene	2.58 × 10^5^	10.47
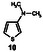 3‐(Dimethylamino)thiophene	9.03 × 10^4^	10.02
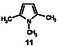 1,2,3‐Trimethylpyrrole	2.40 × 10^4^	9.44
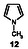 *N*‐Methylpyrrole	1.00 × 10^2^	7.06
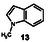 *N*‐Methylindole	3.51 × 10^3^	8.61
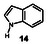 Indole	5.55 × 10^2^	7.80

a) Second‐order rate constants *k* were taken from ref. [1].

b) Nucleophilicity parameters *N* were estimated in this work from Equation (4) by using *k*, *E*, and s_N_  = 1.

c) Experimental *N* value determined in this work (Figure 5).

Six nucleophiles were selected, covering a representative set of *π*‐activated C‐nucleophiles, namely pyrroles, indoles, and aminothiophenes (see Table 2 for full structural and kinetic data). As illustrated in Table 2, 3‐aminothiophenes **1**, **9**, and **10** display markedly higher nucleophilic reactivity than both pyrroles **11**–**12** and indoles **13**–**14**. This enhanced reactivity stems from the strong electron‐donating effect of the amino substituent at the 3‐position, which increases the electron density at the adjacent carbon and facilitates the formation of a Meisenheimer‐type *σ*‐complex^[^ ^6^, ^7^, ^8^, ^30^, ^31^, ^32^ ^]^ during nucleophilic attack. In contrast, in pyrroles and indoles, the nitrogen lone pair is delocalized into the aromatic sextet, stabilizing aromaticity but reducing its availability for nucleophilic activation. This delocalization lowers the electron density at the reactive carbons, thus diminishing nucleophilicity. Collectively, these findings confirm the pronounced enamine‐like character of 3‐aminothiophenes, where the amino lone pair conjugates effectively with the thiophene π‐system, strongly enhancing nucleophilic activation at C‐2, as depicted in resonance structures I and II.^[^ ^1^, ^9^ ^]^

### 2.5 Quantitative Prediction of Nucleophilicity and Hyper‐Ortho Reactivity in 3‐Aminothiophenes Using Hammett *σ* ^+^ Constants

**Figure 6:**
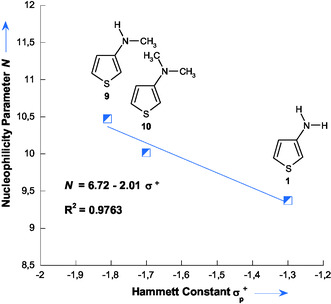
Correlation of *N* values with Hammett *σ* ^+^ constants for aminothiophenes **1**, **9**, and **10** in 50% H_2_O–50% Me_2_SO v/v, highlighting the enaminic behavior and the hyper‐ortho relationship.

A significant feature emerging from Table 2 is the behavior of the three 3‐substituted aminothiophenes, 3‐aminothiophene **1** (*X* = NH_2_), 3‐(dimethylamino)thiophene **10** (*X* = N(CH_3_)_2_), and 3‐(methylamino)thiophene **9** (*X* = NHCH_3_), which possess substituents at the 3‐position with progressively increasing electron‐donating abilities. This trend is reflected in their decreasing constants Hammett (*σ* ^+^), which are −1.30 for NH_2_, −1.70 for N(CH_3_)_2_, and –1.81 for NHCH_3_, respectively.^[^ ^33^ ^]^ The systematic variation in electronic properties is directly correlated with their experimental nucleophilicity parameters (*N*): 9.37 for compound **1**, 10.02 for **10**, and 10.47 for **9**. The direct relationship between the nucleophilicity *N* of 3‐aminothiophenes and their *σ* ^+^ constants is illustrated in **Figure** **6**, where *N* is plotted as a function of *σ* ^+^. A good linear correlation is observed and is described by Equation (6).

(6) $$N=6.72-2.01\;\sigma^{+}\;\;\;(R^{2}=0.9763)$$

This high correlation coefficient (*R* ^2^ = 0.9763) emphasizes the strong influence of the substituent's electronic effects on nucleophilicity. Moreover, this correlation not only highlights the pronounced enaminic behavior of 3‐aminothiophene^[^ ^1^, ^9^, ^13^ ^]^ but also suggests that the nucleophilic reactivity within this series is governed by a hyper‐ortho electronic effect,^[^ ^34^, ^35^ ^]^ whereby substituent–*π*‐system interactions at the 3‐position play a decisive role.

This empirical relationship further enabled the extrapolation of *N* values for structurally related 3‐substituted thiophenes for which no direct kinetic measurements are available. Using the Hammett *σ* ^+^ constants of –0.78, –0.31, and 0.00^[^ ^33^ ^]^ for thiophenes **15** (OCH_3_), **16** (CH_3_), and **17** (H), the corresponding *N* values were estimated as 8.29, 7.34, and 6.72 in 50% H_2_O–50% Me_2_SO v/v.


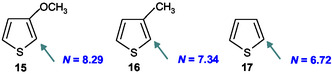


These predictions demonstrate the quantitative applicability of the *N* versus *σ* ^+^ relationship within the thiophene series and further support the hypothesis that substituent effects at the 3‐position directly modulate nucleophilic reactivity through electronic delocalization and resonance donation into the *π*‐system of the thiophene ring.

The linear correlation observed here between the nucleophilicity parameter (*N*) and the Hammett *σ* ^+^ constants for 3‐substituted thiophenes is consistent with findings reported for other aromatic and heterocyclic compounds.^[^ ^36^, ^37^, ^38^ ^]^ For instance, Gabsi and coworkers reported a strong linear relationship between *N* and *σ*_p_^−^ values for *para*‐substituted phenoxide anions in water (*N* = 9.21 − 2.51*σ*_p_^−^ ; *R* ^2^ = 0.9763).^[^ ^37^ ^]^ Similarly, Ghabi and coworkers showed that the nucleophilicity of *para*‐substituted anilines in methanol correlates well with their *σ*_p_ constants (*N* = 12.46 – 5.89 *σ*_p_ ; *R* ^2^ = 0.9982).^[^ ^36^ ^]^

These examples support the general idea that electronic substituent effects, as measured by Hammett constants, can be used to predict and compare nucleophilic reactivity in a wide range of systems. Our results with aminothiophenes add to this growing body of evidence and confirm the usefulness of such correlations for understanding and anticipating structure–reactivity relationships.

It is noteworthy that the nucleophilicity parameters *N* of thiophenes **15–17** are applicable for predicting rate constants of their reactions with other electrophiles. Kinetic data for the reactions of **15–17** with 4,6‐dinitrobenzotriazole **18** ^[^ ^38^, ^39^ ^]^ in 50% H_2_O‐50% Me_2_SO v/v at 20 °C are summarized in **Table** **3** and detailed in Table S12 and Figure S10, Supporting Information confirming this applicability.

**Table 3:** Experimental and calculated second‐order rate constants for the reactions of 4,6‐dinitrobenzotriazole 18 with a series of thiophenes 15‐17 in 50% H_2_O–50% Me_2_SO v/v at 20 °C.

Electrophile	Nucleophile	*N* b)	Rate constants [mol^−1^ L s^−1^]		
			*k*_1_^exp^ b)	*k*_1_^calcd^ c)	*k*_1_^exp^/*k*_1_^calcd^
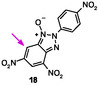 *E* = −9.56a)	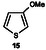	8.29	4.51 × 10^−1^	5.37 × 10^−2^	8.4
	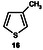	7.34	9.38 × 10^−2^	6.03 × 10^−3^	15.6
	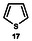	6.72	1.60 × 10^−2^	1.46 × 10^−3^	11.1

a) Electrophilicity parameter *E* value taken from Ref. [38, 39]

b) This work.

c) *k*_1_^calcd^ values calculated using Equation (4) with the nucleophilicity parameters *N* (and s_N_ = 1) of thiophenes **15–17** in 50% H_2_O–50% Me_2_SO v/v as given in this Table, and the electrophilicity parameter *E* of 4,6‐dinitrobenzotriazole **18**.

As shown in Table 3, the predicted and experimental rate constants remain within a factor of 8.4–15.6, which falls well inside the confidence limits of Mayr's approach (factor 10–100).^[^ ^40^, ^41^, ^42^ ^]^ This validation strongly supports the use of these *N* values, together with those of thiophenes **1**, **9**, and **10**, as a reliable reference set for quantifying the reactivity of other electrophiles, including super‐electrophiles.

## 3 Conclusion

The electrophilic substitution occurs at the C–2 position of 3–aminothiophene, and the rate‐determining step is not influenced by deuterium substitution at C–3, confirming a classical S_E_Ar mechanism. The curvature observed in the Hammett plot is attributed to the pronounced resonance effects exerted by electron‐donating substituents. This interpretation is supported by the Yukawa–Tsuno analysis (*ρ* = 2.11, *r* = 1.54), which underscores the dominant role of resonance stabilization over inductive contributions in governing the reactivity of substituted diazonium cations. 3–Aminothiophene exhibits high nucleophilicity (*N* = 9.37 and s_N_ = 1.18) in 50% H_2_O–50% Me_2_SO v/v, comparable to conjugated enamines, and reacts rapidly with a range of electrophiles in *π*‐type S_E_Ar reactions. A strong linear relationship was found between the nucleophilicity N and the Hammett *σ* ^+^ constants of substituents at the C‐3 position of 3‐aminothiophenes, expressed as *N* = 6.72 – 2.01 *σ* ^+^. This equation highlights the role of hyper ortho electronic effects, i.e., direct substituent–*π*‐system interactions, on nucleophilic reactivity and reinforces the enaminic nature of 3‐aminothiophenes. The *N* vs. *σ* ^+^ correlation enables extrapolation to other 3‐substituted thiophenes. For example, predicted *N* values are 8.29 (OCH_3_), 7.34 (CH_3_), and 6.72 (H), consistent with their electronic properties. This predictive model is useful for designing nucleophilic heterocycles in synthetic and mechanistic studies.

## 4 Experimental Section

4.1

4.1.1

##### Materials

3‐Aminothiophene **1** used in this study was prepared and characterized according to a previously reported procedure.^[^ ^1^, ^9^ ^]^

All *para*‐R‐substituted benzenediazonium tetrafluoroborate salts (*para*‐R‐C_6_H_4_N_2_^+^·BF_4−_) **7a**–**7h** were freshly prepared by diazotization of the corresponding *para*‐R‐anilines in a 50% aqueous solution of fluoroboric acid (HBF_4_) at 0 °C, as described in the literature.^[^ ^15^, ^43^ ^]^

Dimethyl sulfoxide (Me_2_SO, Aldrich, ≥99.9%, HPLC grade) was used as received, without further purification.

##### Kinetics Measurements

Kinetic measurements were conducted using a Shimadzu UV‐Vis spectrophotometer (Model 1650) and a Biologic Stopped‐Flow Spectrophotometer (Model SFM‐X00/Q). Temperature control was ensured using a thermoelectrically regulated cell holder (Model TCC‐240 A), maintaining a stable temperature of 20.0 ± 0.1 °C. Pseudo‐first‐order rate constants *k*_obsd_ were calculated using Equation (7),^[^ ^1^, ^13^, ^15^ ^]^ where *A*_∞_ refers to the absorbance measured at the equilibrium state of the reaction between diazonium salt **7a–7h** and aminothiophene **1**, *A*_o_ refers to the absorbance at zero time, and ***A***_t_ refers to the absorbance at time t. Correlation coefficients of the linear regressions were usually higher than 0.97. The variations of the observed rate constants *k*_obsd_ as a function of the concentrations of the carboxylic acids are shown in Figure S1–S9 and Table S1–S11, Supporting Information.

(7) $$\text{ln}\;\left(A_{\infty }-A_{t}\right)\;=\;-k_{\text{obsd}}t\;+\;ln\;\left(A_{\infty }-A_{o}\right)$$

## Supporting Information

**Figures S1–S6:** Effects of the total concentration of 3‐aminothiophene **1** and pH on the pseudo‐first‐order rate constants (*k*_obsd_) for its addition reactions with benzenediazonium salts **7a–7c** and **7f–7h** in 50% H_2_O–50% Me_2_SO (v/v) mixture at 20 °C. Measurements were conducted at pH 1 and 2.

**Figures S7–S9:** Effects of the total concentration of 2‐deuterio‐3‐aminothiophene and pH on the pseudo‐first‐order rate constants (*k*_obsd_) for its addition reactions with benzenediazonium salts **7a, 7c** and **7g** in 50% H_2_O–50% Me_2_SO (v/v) at 20 °C. Measurements were conducted at pH 1 and 2.

**Tables S1–S8:** Concentrations and observed rate constants (kobsd) from individual kinetic experiments of the reactions between 3‐aminothiophene **1** and benzenediazonium salts **7a–7h** in 50% H_2_O–50% Me_2_SO (v/v) mixture at 20 °C.

**Tables S9–S11:** Concentrations and observed rate constants (*k*_obsd_) from individual kinetic experiments of the reactions between 2‐deuterio‐3‐aminothiophene and benzenediazonium salts **7a, 7c** and **7g** in 50% H_2_O–50% Me_2_SO (v/v) at 20 °C.

Kinetic studies: Reaction of 4,6‐dinitrobenzotriazole **18** with thiophenes **15–17** in 50% H_2_O–50% Me_2_SO (v/v) mixture at 20 °C.

## Conflict of Interest

The authors declare no conflict of interest.

## Author Contributions

**R. El Abed**: data curation (equal); investigation (lead); and writing—original draft (equal). **T. Slama**: data curation (equal); software (equal); and visualization (equal). **F. Mahdhaoui**: data curation (equal); software (equal); and visualization (equal). **T. Boubaker**: conceptualization (equal); investigation (equal); supervision (lead); and writing—review and editing (equal).

## Supporting information

Supplementary Material

## Data Availability

The data that support the findings of this study are available in the supplementary material of this article.
