# Supplementary material for: C–N Coupling of 3‐Aminothiophene with Substituted Benzenediazonium Cations: Carbon Nucleophilicity, Hyper‐Ortho Effects, and Predictive Modeling of Structural Analogs
Source: Chemphyschem. 2025 Nov 3;26(24):e202500553. doi: 10.1002/cphc.202500553 (PMC12710183; doi:10.1002/cphc.202500553)

Supporting Information

C–N Coupling of 3-Aminothiophene with Substituted Benzenediazonium Cations: Carbon Nucleophilicity, Hyper-Ortho Effects, and Predictive Modelling of Structural Analogues

Riadh El Abed, Takwa Slama, Foauzi Mahdhaoui and Taoufik Boubaker*

Laboratory of Heterocyclic Chemistry, Natural Products, and Reactivity (LR11SE39), Faculty of Sciences of Monastir, University of Monastir, Avenue of the Environment, 5019 Monastir, Tunisia.

Table of Contents

Section 1 (pp. 2–4)
Effects of the total concentration of 3-aminothiophene **1** and pH on the pseudo-first-order rate constants (k_{obsd}) for its addition reactions with benzenediazonium salts **7a–7c** and **7f–7h** in 50% H_2O–50% Me_2SO (v/v) at 20 °C. Measurements were conducted at pH 1 and 2 (Figures S1–S6).

Section 2 (pp. 5–6)
Effects of the total concentration of 2-deuterio-3-aminothiophene and pH on the pseudo-first-order rate constants (k_{obsd}) for its addition reactions with benzenediazonium salts **7a**, **7c** and **7g** in 50% H_2O–50% Me_2SO (v/v) at 20 °C. Measurements were conducted at pH 1 and 2 (Figures S7–S9).

Section 3 (pp. 7–10)
Concentrations and observed rate constants (k_{obsd}) from individual kinetic experiments of the reactions between 3-aminothiophene **1** and benzenediazonium salts **7a–7h** in 50% H_2O–50% Me_2SO (v/v) at 20 °C (Tables S1–S8).

Section 4 (pp. 11)
Concentrations and observed rate constants (k_{obsd}) from individual kinetic experiments of the reactions between 2-deuterio-3-aminothiophene and benzenediazonium salts **7a**, **7c** and **7g** in 50% H_2O–50% Me_2SO (v/v) at 20 °C (Tables S9–S11).

Section 5 (pp. 12-13)
Kinetic studies: Reaction of 4,6-dinitrobenzotriazole **18** with thiophenes **15–17** in 50% H_2O–50% Me_2SO (v/v) at 20 °C.

Section 1 (pp. 2–4)

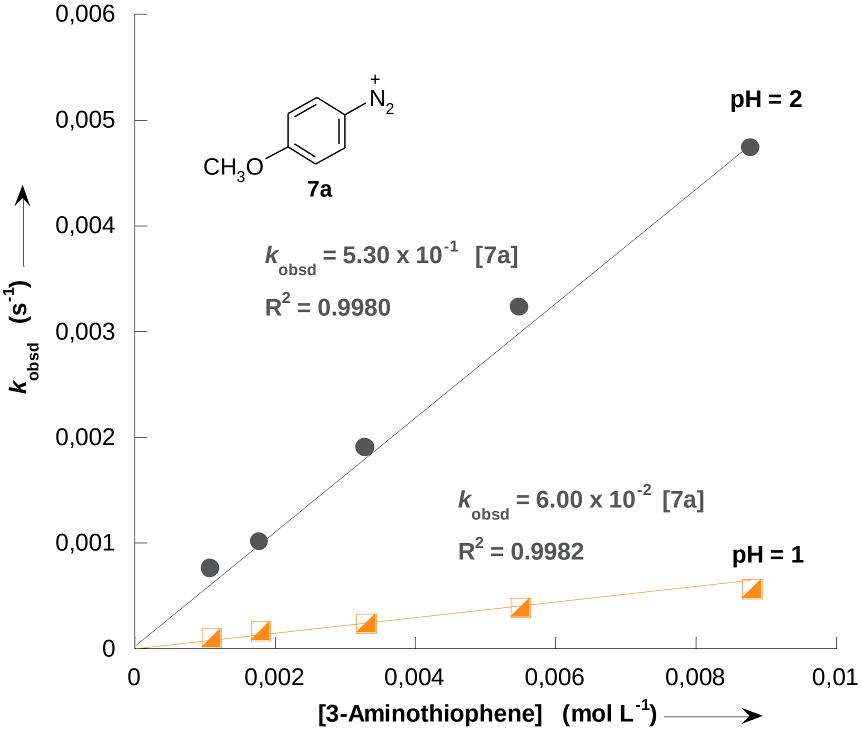

Figure S1. Influence of the total concentration of 3-aminothiophene **1** on the pseudo-first-order rate constants (k_{obsd}) for its addition reactions with benzenediazonium salt **7a** in 50% H_2O–50% Me_2SO (v/v) at 20 °C.

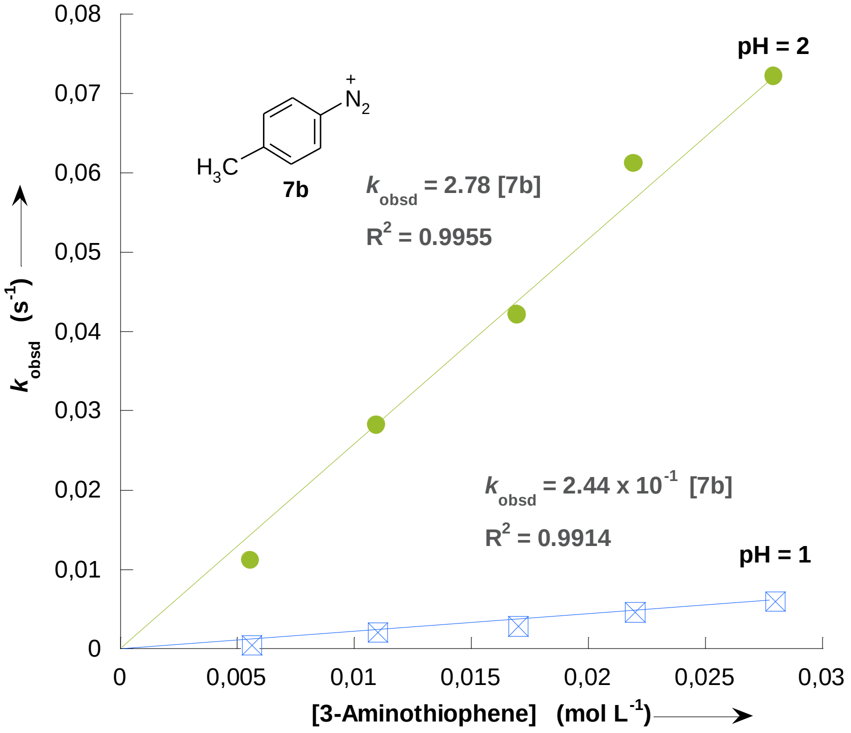

Figure S2. Influence of the total concentration of 3-aminothiophene **1** on the pseudo-first-order rate constants (k_{obsd}) for its addition reactions with benzenediazonium salt **7b** in 50% H_2O–50% Me_2SO (v/v) at 20 °C.

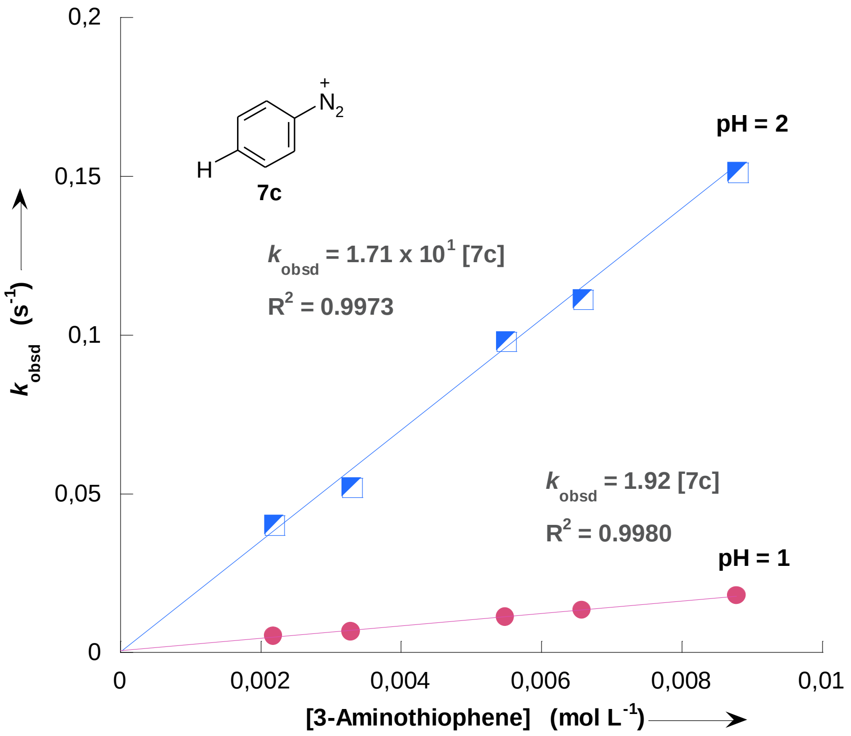

Figure S3. Influence of the total concentration of 3-aminothiophene **1** on the pseudo-first-order rate constants (k_{obsd}) for its addition reactions with benzenediazonium salt **7c** in 50% H_2O–50% Me_2SO (v/v) at 20 °C.

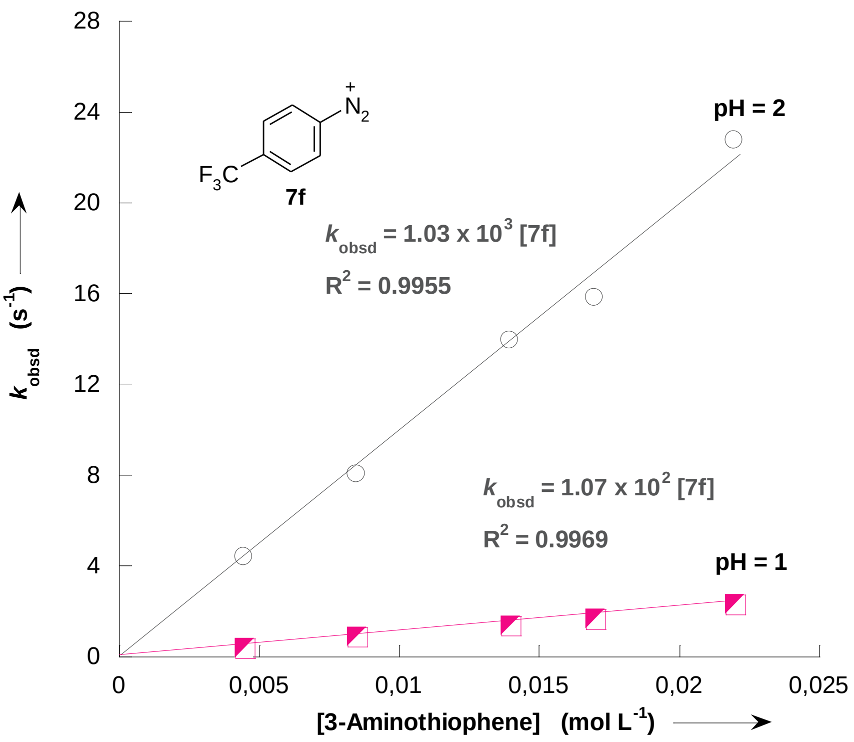

Figure S4. Influence of the total concentration of 3-aminothiophene **1** on the pseudo-first-order rate constants (k_{obsd}) for its addition reactions with benzenediazonium salt **7f** in 50% H_2O–50% Me_2SO (v/v) at 20 °C.

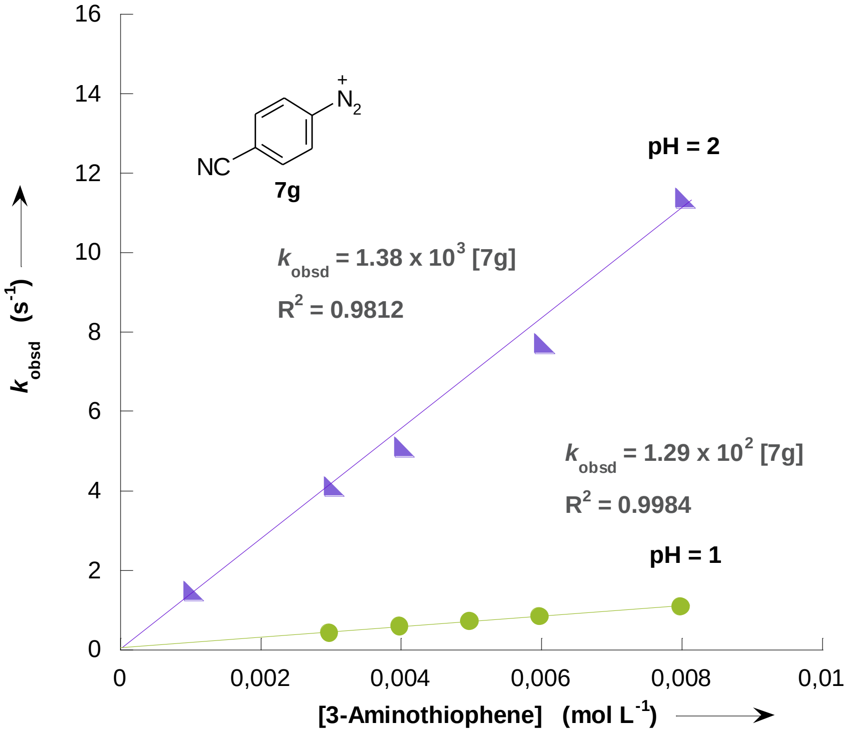

Figure S5. Influence of the total concentration of 3-aminothiophene **1** on the pseudo-first-order rate constants (k_{obsd}) for its addition reactions with benzenediazonium salt **7g** in 50% H_2O–50% Me_2SO (v/v) at 20 °C.

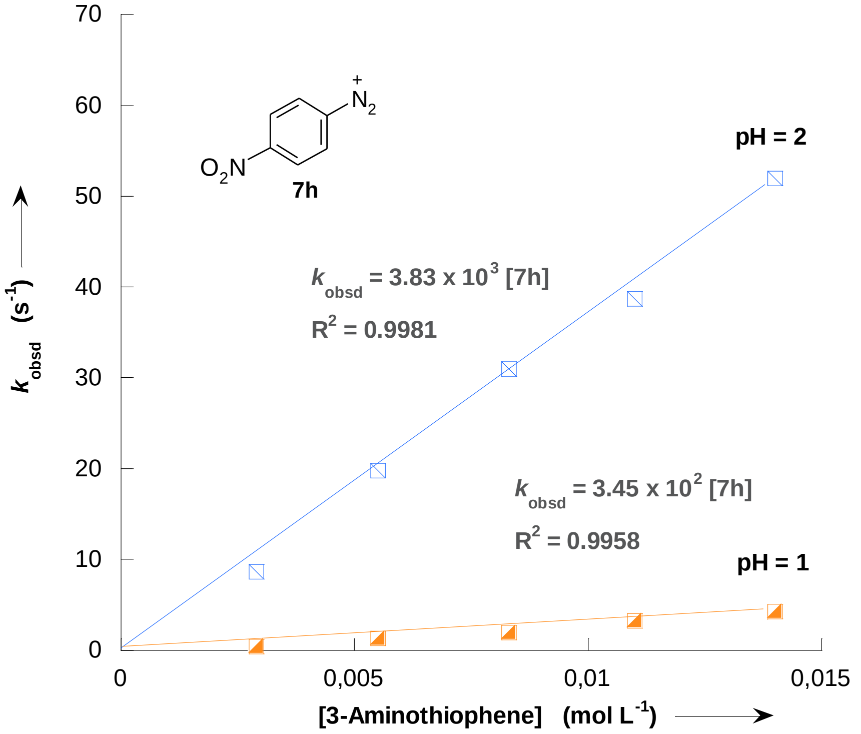

Figure S6. Influence of the total concentration of 3-aminothiophene **1** on the pseudo-first-order rate constants (k_{obsd}) for its addition reactions with benzenediazonium salt **7h** in 50% H_2O–50% Me_2SO (v/v) at 20 °C.

Section 2 (pp. 5–6)

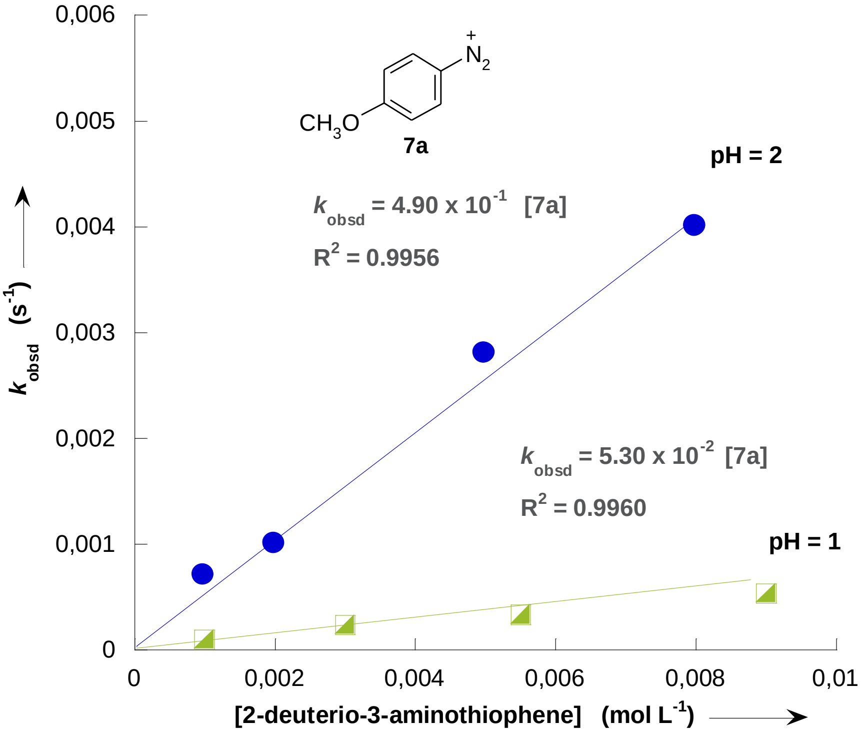

Figure S7. Influence of the total concentration of 2-deuterio-3-aminothiophene on the pseudo-first-order rate constants (k_{obsd}) for its addition reactions with benzenediazonium salt **7a** in 50% H_2O–50% Me_2SO (v/v) at 20 °C.

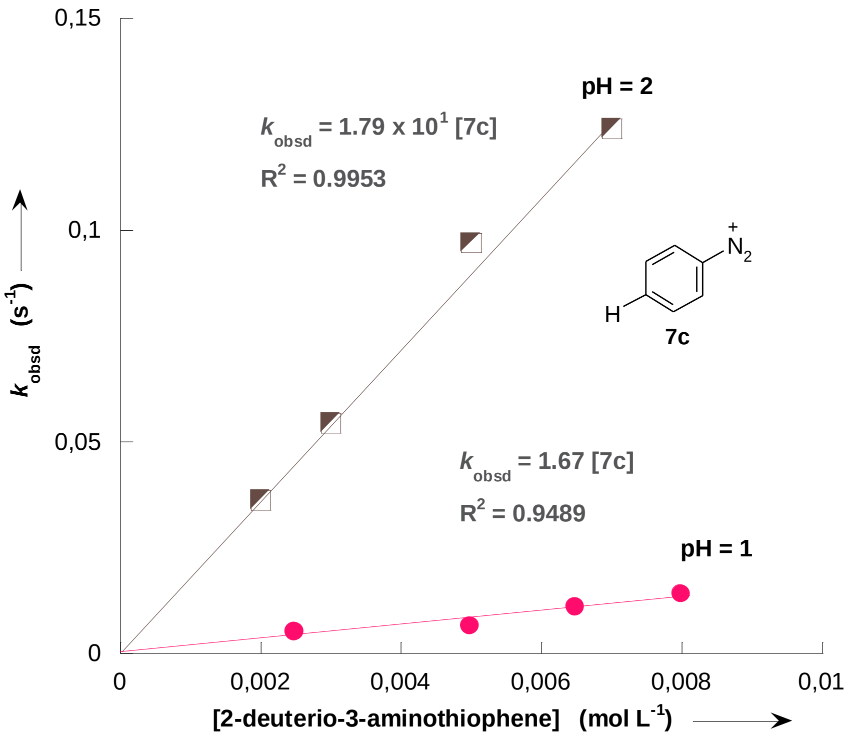

Figure S8. Influence of the total concentration of 2-deuterio-3-aminothiophene on the pseudo-first-order rate constants (k_{obsd}) for its addition reactions with benzenediazonium salt **7c** in 50% H_2O–50% Me_2SO (v/v) at 20 °C.

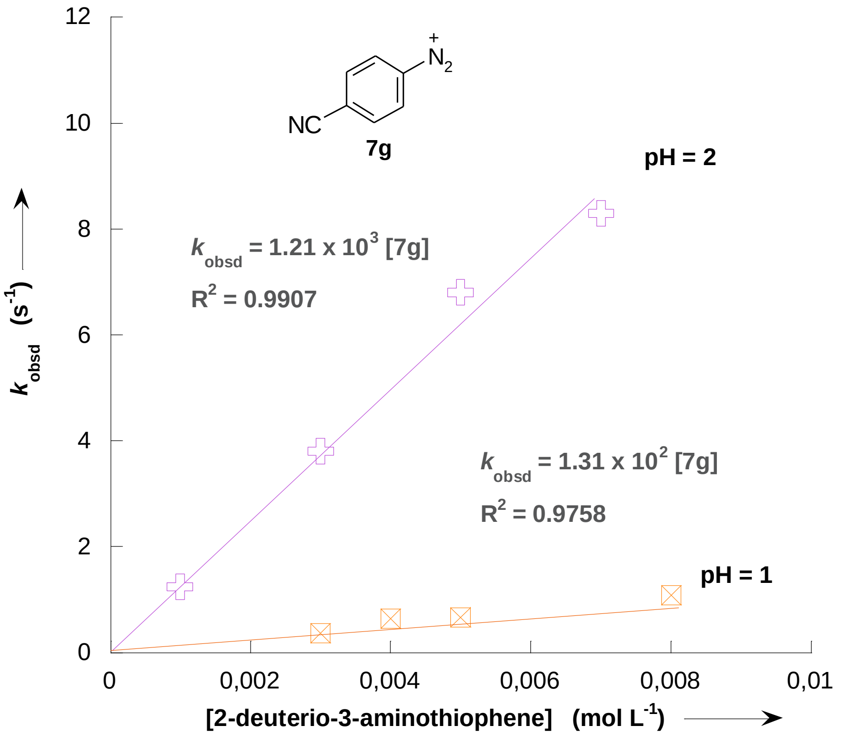

Figure S9. Influence of the total concentration of 2-deuterio-3-aminothiophene on the pseudo-first-order rate constants (k_{obsd}) for its addition reactions with benzenediazonium salt **7g** in 50% H_2O–50% Me_2SO (v/v) at 20 °C.

Section 3 (pp. 7–10)

Table S1. The k_{obsd} and k values for the reactions of the 3-aminothiophene **1** with benzenediazonium salt **7a** in 50% H_2O–50% Me_2SO (v/v) at 20 °C.

Nucleophile	pH	[3-Aminothiophene] mol L^{-1}	k_{obsd} s^{-1}	k mol^{-1} L s^{-1}	Correlation coefficient
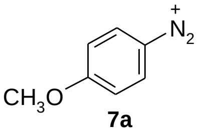 **7a**	2	1.10×10^{-3}<br>1.80×10^{-3}<br>3.30×10^{-3}<br>5.50×10^{-3}<br>8.80×10^{-3}	7.50×10^{-4}<br>1.00×10^{-3}<br>1.89×10^{-3}<br>3.22×10^{-3}<br>4.72×10^{-3}	5.30×10^{-1}	0.9980
	1	1.10×10^{-3}<br>1.80×10^{-3}<br>3.30×10^{-3}<br>5.50×10^{-3}<br>8.80×10^{-3}	9.90×10^{-5}<br>1.65×10^{-4}<br>2.38×10^{-4}<br>3.86×10^{-4}<br>5.65×10^{-4}	6.00×10^{-2}	0.9982

Table S2. The k_{obsd} and k values for the reactions of the 3-aminothiophene **1** with benzenediazonium salt **7b** in 50% H_2O–50% Me_2SO (v/v) at 20 °C.

Nucleophile	pH	[3-Aminothiophene]<br>mol L^{-1}	k_{obsd}<br>s^{-1}	k<br>mol^{-1} L s^{-1}	Correlation coefficient
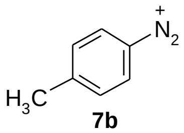<br>**7b**	2	5.60×10^{-3}<br>1.10×10^{-2}<br>1.70×10^{-2}<br>2.20×10^{-2}<br>2.80×10^{-2}	1.10×10^{-2}<br>2.80×10^{-2}<br>4.20×10^{-2}<br>6.10×10^{-2}<br>7.20×10^{-2}	2.78	0.9955
	1	5.60×10^{-3}<br>1.10×10^{-2}<br>1.70×10^{-2}<br>2.20×10^{-2}<br>2.80×10^{-2}	4.40×10^{-4}<br>2.10×10^{-3}<br>2.80×10^{-3}<br>4.60×10^{-3}<br>6.00×10^{-3}	2.44×10^{-1}	0.9914

Table S3. The k_{obsd} and k values for the reactions of the 3-aminothiophene **1** with benzenediazonium salt **7c** in 50% H_2O–50% Me_2SO (v/v) at 20 °C.

Nucleophile	pH	[3-Aminothiophene]<br>mol L^{-1}	k_{obsd}<br>s^{-1}	k<br>mol^{-1} L s^{-1}	Correlation coefficient
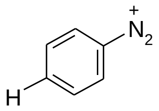<br>**7c**	2	2.20×10^{-3}<br>3.30×10^{-3}<br>5.50×10^{-3}<br>6.60×10^{-3}<br>8.80×10^{-3}	4.00×10^{-2}<br>5.20×10^{-2}<br>9.79×10^{-2}<br>1.11×10^{-1}<br>1.51×10^{-1}	1.71×10^{1}	0.9973
	1	2.20×10^{-3}<br>3.30×10^{-3}<br>5.50×10^{-3}<br>6.60×10^{-3}<br>8.80×10^{-3}	4.90×10^{-3}<br>6.20×10^{-3}<br>1.08×10^{-2}<br>1.28×10^{-2}<br>1.74×10^{-2}	1.92	0.9980

Table S4. The k_{obsd} and k values for the reactions of the 3-aminothiophene **1** with benzenediazonium salt **7d** in 50% H_2O–50% Me_2SO (v/v) at 20 °C.

Nucleophile	pH	[3-Aminothiophene]<br>mol L^{-1}	k_{obsd}<br>s^{-1}	k<br>mol^{-1} L s^{-1}	Correlation coefficient

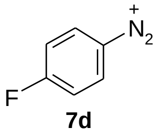 **7d**	2	3.00 x 10^{-3}<br>4.00 x 10^{-3}<br>5.00 x 10^{-3}<br>6.00 x 10^{-3}<br>7.00 x 10^{-3}	1.32 x 10^{-1}<br>1.82 x 10^{-1}<br>2.02 x 10^{-1}<br>2.38 x 10^{-1}<br>3.10 x 10^{-1}	4.12 x 10^{1}	0.9799
	1	3.00 x 10^{-3}<br>4.00 x 10^{-3}<br>5.00 x 10^{-3}<br>6.00 x 10^{-3}<br>7.00 x 10^{-3}	1.13 x 10^{-2}<br>1.64 x 10^{-2}<br>1.87 x 10^{-2}<br>2.49 x 10^{-2}<br>2.80 x 10^{-2}	4.19	0.9924

Table S5. The k_{obsd} and k values for the reactions of the 3-aminothiophene **1** with benzenediazonium salt **7e** in 50% H_2O–50% Me_2SO (v/v) at 20 °C.

Nucleophile	pH	[3-Aminothiophene] mol L^{-1}	k_{obsd} s^{-1}	k mol^{-1} L s^{-1}	Correlation coefficient
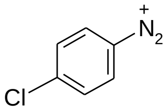 **7e**	2	2.00 x 10^{-3}<br>4.00 x 10^{-3}<br>5.00 x 10^{-3}<br>6.00 x 10^{-3}<br>8.00 x 10^{-3}	1.52 x 10^{-1}<br>3.34 x 10^{-1}<br>3.81 x 10^{-1}<br>5.20 x 10^{-1}<br>6.54 x 10^{-1}	8.46 x 10^{1}	0.9941
	1	2.00 x 10^{-3}<br>4.00 x 10^{-3}<br>5.00 x 10^{-3}<br>6.00 x 10^{-3}<br>8.00 x 10^{-3}	1.73 x 10^{-2}<br>3.05 x 10^{-2}<br>4.10 x 10^{-2}<br>4.92 x 10^{-2}<br>6.80 x 10^{-2}	8.54	0.9968

Table S6. The k_{obsd} and k values for the reactions of the 3-aminothiophene **1** with benzenediazonium salt **7f** in 50% H_2O–50% Me_2SO (v/v) at 20 °C.

Nucleophile	pH	[3-Aminothiophene] mol L^{-1}	k_{obsd} s^{-1}	k mol^{-1} L s^{-1}	Correlation coefficient

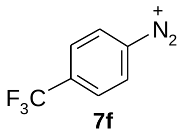<br>**7f**	2	4.40×10^{-3}<br>8.50×10^{-3}<br>1.40×10^{-2}<br>1.70×10^{-2}<br>2.20×10^{-2}	4.35<br>8.01<br>1.39×10^{1}<br>1.58×10^{1}<br>2.27×10^{1}	1.03×10^{3}	0.9955
	1	4.40×10^{-3}<br>8.50×10^{-3}<br>1.40×10^{-2}<br>1.70×10^{-2}<br>2.20×10^{-2}	3.40×10^{-1}<br>8.62×10^{-1}<br>1.34<br>1.63<br>2.28	1.07×10^{2}	0.9969

Table S7. The k_{obsd} and k values for the reactions of the 3-aminothiophene **1** with benzenediazonium salt **7g** in 50% H_2O–50% Me_2SO (v/v) at 20 °C.

Nucleophile	pH	[3-Aminothiophene]<br>mol L^{-1}	k_{obsd}<br>s^{-1}	k<br>mol^{-1} L s^{-1}	Correlation coefficient
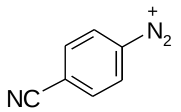<br>**7g**	2	1.00×10^{-3}<br>3.00×10^{-3}<br>4.00×10^{-3}<br>6.00×10^{-3}<br>8.00×10^{-3}	4.10<br>5.10<br>1.48<br>7.70<br>1.14×10^{1}	1.38×10^{3}	0.9812
	1	3.00×10^{-3}<br>4.00×10^{-3}<br>5.00×10^{-3}<br>6.00×10^{-3}<br>8.00×10^{-3}	3.90×10^{-1}<br>5.58×10^{-1}<br>6.69×10^{-1}<br>7.94×10^{-1}<br>1.05	1.29×10^{2}	0.9984

Table S8. The k_{obsd} and k values for the reactions of the 3-aminothiophene **1** with benzenediazonium salt **7h** in 50% H_2O–50% Me_2SO (v/v) at 20 °C.

Nucleophile	pH	[3-Aminothiophene]<br>mol L^{-1}	k_{obsd}<br>s^{-1}	k<br>mol^{-1} L s^{-1}	Correlation coefficient

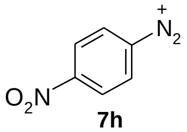<br>**7h**	2	2.90×10^{-3}<br>5.50×10^{-3}<br>8.30×10^{-3}<br>1.10×10^{-2}<br>1.40×10^{-2}	8.65<br>1.97×10^{1}<br>3.10×10^{1}<br>3.87×10^{1}<br>5.20×10^{1}	3.83×10^{3}	0.9981
	1	2.90×10^{-3}<br>5.50×10^{-3}<br>8.30×10^{-3}<br>1.10×10^{-2}<br>1.40×10^{-2}	4.13×10^{-1}<br>1.27<br>1.95<br>3.20<br>4.22	3.45×10^{2}	0.9958

Section 4 (pp. 11)

Table S9. The k_{obsd} and k values for the reactions of the 2-deuterio-3-aminothiophene with benzenediazonium salt **7a** in 50% H_2O–50% Me_2SO (v/v) at 20 °C.

Nucleophile	pH	[3-Aminothiophene]<br>mol L^{-1}	k_{obsd}<br>s^{-1}	k<br>mol^{-1} L s^{-1}	Correlation coefficient
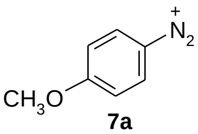<br>**7a**	2	1.00×10^{-3}<br>2.00×10^{-3}<br>5.00×10^{-3}<br>8.00×10^{-3}	7.00×10^{-4}<br>1.03×10^{-3}<br>2.80×10^{-3}<br>4.02×10^{-3}	4.90×10^{-1}	0.9956
	1	1.00×10^{-3}<br>3.00×10^{-3}<br>5.50×10^{-3}<br>9.00×10^{-3}	9.87×10^{-5}<br>2.38×10^{-4}<br>3.34×10^{-4}<br>5.35×10^{-4}	5.30×10^{-2}	0.9960

Table S10. The k_{obsd} and k values for the reactions of the 2-deuterio-3-aminothiophene with benzenediazonium salt **7c** in 50% H_2O–50% Me_2SO (v/v) at 20 °C.

Nucleophile	pH	[3-Aminothiophene]<br>mol L^{-1}	k_{obsd}<br>s^{-1}	k<br>mol^{-1} L s^{-1}	Correlation coefficient

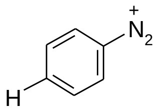 **7c**	2	2.00 x 10^{-3}<br>3.00 x 10^{-3}<br>5.00 x 10^{-3}<br>7.00 x 10^{-3}	3.62 x 10^{-2}<br>5.44 x 10^{-2}<br>9.70 x 10^{-2}<br>1.24 x 10^{-1}	1.79 x 10^{1}	0.9953
	1	2.50 x 10^{-3}<br>5.00 x 10^{-3}<br>6.50 x 10^{-3}<br>8.00 x 10^{-3}	4.90 x 10^{-3}<br>6.20 x 10^{-3}<br>1.08 x 10^{-2}<br>1.38 x 10^{-2}	1.67	0.9489

Table S11. The k_{obsd} and k values for the reactions of the 2-deuterio-3-aminothiophene with benzenediazonium salt **7g** in 50% H_2O–50% Me_2SO (v/v) at 20 °C.

Nucleophile	pH	[3-Aminothiophene] mol L^{-1}	k_{obsd} s^{-1}	k mol^{-1} L s^{-1}	Correlation coefficient
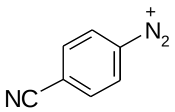 **7g**	2	1.00 x 10^{-3}<br>3.00 x 10^{-3}<br>5.00 x 10^{-3}<br>7.00 x 10^{-3}	1.24<br>3.80<br>6.80<br>8.30	1.21 x 10^{3}	0.9907
	1	3.00 x 10^{-3}<br>4.00 x 10^{-3}<br>5.00 x 10^{-3}<br>8.00 x 10^{-3}	3.70 x 10^{-1}<br>6.50 x 10^{-1}<br>6.70 x 10^{-1}<br>1.08	1.31 x 10^{2}	0.9758

Section 5 (pp. 12-13)

Kinetic Studies: Reaction of 4,6-dinitrobenzotriazole **18** with thiophenes **15-17** in 50% H_2O–50% Me_2SO (v/v) at 20 °C.

The σ-complexation reactions of 4,6-dinitrobenzotriazole **18** with thiophenes **15–17** were kinetically investigated in 50% H_2O-50% Me_2SO (v/v) at 20 °C. In all cases, a single relaxation time corresponding to the quantitative formation of the σ-adducts **19** (463 nm < λ_{max} < 476 nm) was observed. The reactions were performed under pseudo-first-order conditions, with thiophene concentrations (10^{-2} mol L^{-1}) in large excess over that of **18** (5 x 10^{-5} mol L^{-1}). The observed first-order rate constants (k_{obsd}) were obtained from the half-life ($t_{1/2}$) directly estimated from the kinetic traces in Figure S10. The corresponding values of k_{obsd}, $t_{1/2}$, and the derived second-order rate constants (k_1^{exp}) for thiophenes **15–17** are summarized in Table S12.

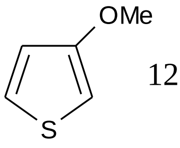

15

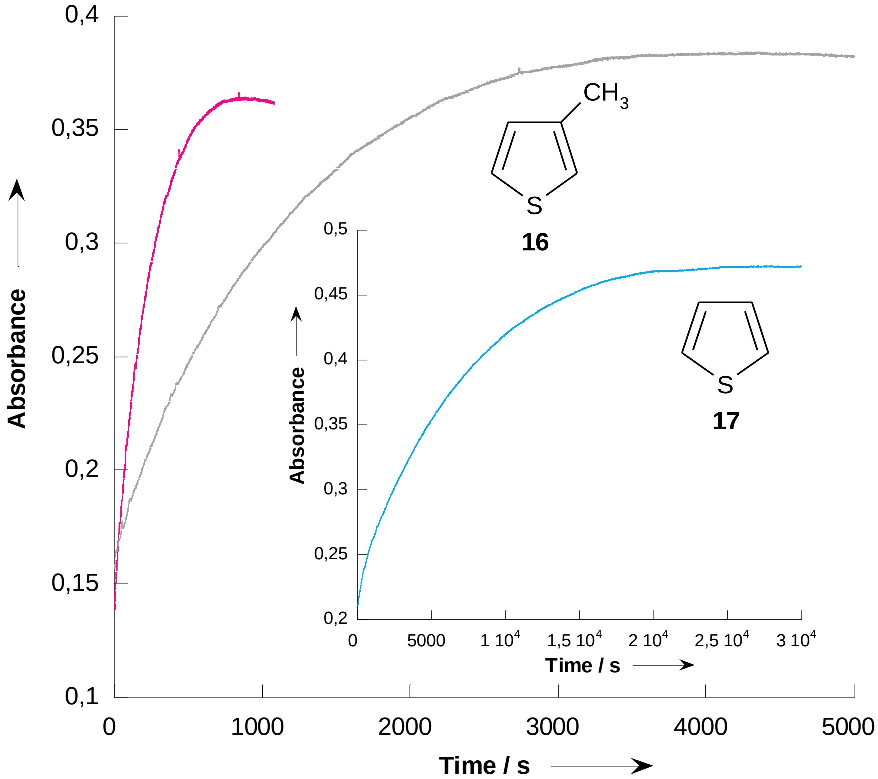

Figure S10. Plot of the absorbance versus time observed for the reaction of 4,6-dinitrobenzotriazole **18** (5 x 10^{-5} mol L^{-1}) with thiophenes **15-17** (10^{-2} mol L^{-1}) in 50% H_2O–50% Me_2SO (v/v) at 20 °C.

Table S12. Kinetic parameters for the σ-complexation reactions of 4,6-dinitrobenzotriazole **18** with thiophenes **15–17** in 50% H_2O–50% Me_2SO (v/v) at 20 °C.

Electrophile	Nucleophile	$t_{1/2}$ [a] (s)	k_{obsd} [b] (s^{-1})	k_1^{exp} [c] (mol^{-1} L s^{-1})
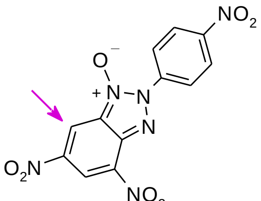 **18**	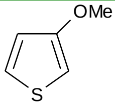 **15**	153	4.51 x 10^{-3}	4.51 x 10^{-1}
	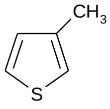 **16**	736	9.38 x 10^{-4}	9.38 x 10^{-2}
	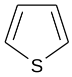 **17**	4313	1.60 x 10^{-4}	1.60 x 10^{-2}

[a] $t_{1/2}$ values are determined from absorbance-time traces (see Figure S7).
[b] k_{obsd} values calculated using k_{obsd} = Ln2 / $t_{1/2}$.

[c] k_1^{exp} values determined using $k_1^{exp} = k_{obsd}$ / [Thiophene].

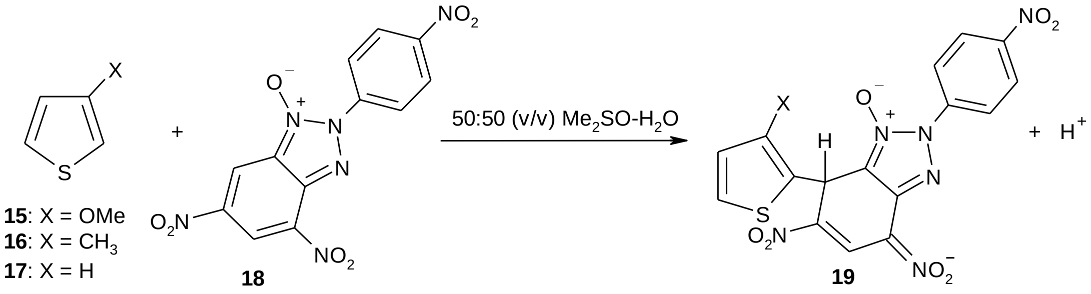

Supplement: Supplementary file 1 — Supplementary Material [file CPHC-26-e202500553-s001.pdf]
